# Virtual Nursing Intervention Adjunctive to Conventional Care: The Experience of Persons Living With HIV

**DOI:** 10.2196/resprot.4158

**Published:** 2015-10-20

**Authors:** José Côté, Geneviève Rouleau, Pilar Ramirez-Garcia, Anne Bourbonnais

**Affiliations:** ^1^Research Centre of the Centre Hospitalier de l’Université de MontréalResearch Chair in Innovative Nursing PracticesMontréal, QCCanada; ^2^Faculty of NursingUniversité de MontréalMontreal, QCCanada

**Keywords:** medication adherence, anti-HIV agents, Internet, HIV infections, nursing research, qualitative research, web-based interventions

## Abstract

**Background:**

Persons living with HIV (PLHIV) must adhere optimally to antiretroviral therapy (ART) on a daily basis and for their lifetime to maintain an undetectable viral load, allowing them to preserve their health. Taking advantage of the opportunity that information and communication technologies provide to broaden intervention modalities and intensify clinical follow-up, a virtual nursing intervention consisting of four interactive computer sessions was developed to empower PLHIV to manage their ART and symptoms optimally. Compared with other types of information and communication technologies-assisted interventions such as text messages, HIV Treatment, Virtual Nursing Assistance and Education (VIH-TAVIE) requires a certain degree of active engagement on the part of the user to develop and strengthen the self-management skills to optimize adherence. After the intervention’s impact on ART adherence was measured quantitatively, a qualitative study was undertaken to describe how users experience the intervention. Understanding how PLHIV perceive being assisted asynchronously by a virtual nurse was of particular interest.

**Objective:**

The objective of the study was to explore and describe how PLHIV experience VIH-TAVIE, that is, receiving customized asynchronous accompaniment via a virtual nurse.

**Methods:**

A qualitative study was conducted with 26 PLHIV (20 men, 6 women) who received all four VIH-TAVIE sessions. Participants had been diagnosed with HIV 14 years earlier on average and had been on ART for a mean period of 10 years. The sessions lasted 20-30 minutes each and were received two weeks apart. They are hosted by a virtual nurse who engages the user in a self-management skills-learning process for the purpose of treatment adherence. Semistructured interviews were conducted lasting 30-40 minutes to get participants to share their experience of the intervention through personal stories and what they thought and felt during their participation. Data were analyzed using Miles and Huberman’s method, by performing these three steps: (1) data reduction (data coding, summaries); (2) data display (in tables and text form); and (3) recontextualization of results.

**Results:**

Content analysis yielded five themes regarding how PLHIV experience VIH-TAVIE: (1) exposure to the virtual nursing intervention; (2) virtual nurse humanizes experience of the computer-delivered intervention; (3) learner’s experience of the virtual nursing intervention; (4) perceived benefits following participation in the virtual nursing intervention; and (5) relevance of the virtual nursing intervention in relation to the medication management trajectory.

**Conclusions:**

Analyzing the participants’ experience revealed they found the intervention’s content and format appropriate. To them, the virtual nurse humanized the experience and helped them acquire new skills for achieving optimal ART adherence. Results seem to underscore the importance of offering the intervention to persons who have more problems with drug intake or who are just beginning ART.

## Introduction

### Achieving Optimal Treatment Adherence With Antiretroviral Therapy

The emergence of antiretroviral therapy (ART) turned HIV infection into a chronic disease. The aim of ART is to attain and maintain an undetectable viral load, enabling persons living with HIV (PLHIV) to maintain their health. To this end, daily antiretroviral drug intake must be adhered to throughout one’s lifetime. In a meta-analysis of 84 studies from 20 countries, Ortego [1] showed that only 62% of PLHIV adhered to ART optimally. This suggests a need for intervention to help PLHIV achieve optimal treatment adherence. In this regard, a recent review of interventions aimed at optimizing ART adherence highlighted five types of intervention with evidence-based efficacy: cognitive-behavioral interventions, education, treatment supporters, directly observed therapy, and active adherence reminder devices such as mobile phone text messages [2].

Text message is the most common type of intervention assisted by information and communication technologies (ICT) used to optimize treatment adherence among PLHIV, and the most tested, as reported by Pellowski and Kalichman [3] in their systematic review of ICT-based programs. Moreover, in their recent meta-analysis, Finitsis et al [4] suggested that text message intervention could help sustain ART adherence. However, some researchers, for example [5,6], have developed and tested computer- or Web-delivered educational and cognitive-behavioral interventions in this regard. These are generally complex and long-term customized health programs that patients can access repeatedly at their convenience. For example, Fisher et al [5] tested a Web-based HIV medication adherence intervention called “Life Windows” composed of several educational modules. This intervention is animated by a virtual animated character. There is no mention if this virtual guide is a health care professional. Their protocol analysis revealed a significant increase in self-reported adherence by the group that received the Web-based intervention. Hersch et al [6], for their part, evaluated a Web-based program named “Life-Steps for Managing Medications and Stress”, which was comprised of nine educational and cognitive-behavioral modules. The Web-based program is fully audio narrated and encompasses video vignettes and other interactive components. Though they reported that the adherence rate among participants who received the program decreased slightly, it declined from about 85% to 66% in the control group. Robbins et al [7] tested a computer-based intervention called Masivukeni on antiretroviral adherence and key psychosocial outcomes among 55 nonadherent South African HIV patients. This intervention includes a multimedia component, where a lay counsellor delivers the intervention to help people living with HIV in resource limited settings achieve optimal adherence. The proportion of participants who achieved 80% adherence or greater, based on the clinic-based pill counts, was 67% among Masivukeni participants and 16% among standard of care (SOC) participants at post intervention (around 5 to 6 weeks post baseline). Participants in the intervention group showed significantly more positive attitudes regarding disclosure and medication social support, less social rejection, and better clinic-patient relationships than SOC patients.

### HIV Treatment, Virtual Nursing Assistance and Education

We developed and tested a virtual intervention called HIV Treatment, Virtual Nursing Assistance and Education or *VIH-TAVIE* (from its French version, *Virus de l’immunodéficience humaine - Traitement assistance virtuelle infirmière et enseignement*) to empower PLHIV to manage their ART and symptoms optimally. VIH-TAVIE consists of four interactive computer sessions hosted by a “virtual” nurse who leads the user through a learning process geared toward acquiring the requisite skills for treatment adherence. What distinguishes this intervention from others is the interaction with a “virtual” nurse, a real nurse that supports and coaches PLHIV in an asynchronous way through video. This nursing intervention aims to reproduce the caregiving relationship (a consultation, a relational practice) between nurse and patient. The videos of a nurse are used to humanize the Web (computer)-based intervention, generating the illusion that someone is there to encourage the patients in their treatment-taking behavior, rather than having exclusively scripted or written content or audio media.

The Web-based interventions cited previously [5-7] have a written audio and may have video components, but there are no “real” health care professionals supporting the learning process. At times, there may be an animated character acting as a counsellor, but it is not the equivalent of having a real health care professional that embodies her professional role in a virtual context. Compared with text message interventions, VIH-TAVIE demands a certain degree of active engagement on the part of the user in order to develop and strengthen the self-regulatory skills required to deal with difficult situations as they arise. The intervention was evaluated in a hospital setting as an adjunct to conventional care. The results of this quasi-experimental study comparing the effectiveness of two types of follow-up—conventional versus conventional plus adjunctive virtual (VIH-TAVIE)—in promoting ART adherence among PLHIV revealed that both interventions improved adherence [8].

### Describing the Participants’ Experience

In addition to implementing quantitative approaches to measure the intervention’s impact on ART adherence, we considered it was also important to describe the users experience with such an ICT-assisted intervention. There is little known about the experience of the participant in Web-based interventions designed to foster antiretroviral taking behavior. In the past, interventions such as Fischer [5], Hersch [6], and Robbins [7], did not address this in a qualitative study. In the current study, we were particularly interested in understanding how PLHIV perceived being assisted asynchronously by a virtual nurse. The qualitative design of the study provides insights into the views of participants regarding their interaction with a virtual health professional and about the timing or dosage of the intervention. These participant perspectives may provide leads for effectively implementing the virtual nursing intervention, VIH-TAVIE, on a larger scale.

## Methods

### Study Design

An exploratory qualitative study based on conventional content analysis [9] was chosen to describe how PLHIV experienced the virtual nursing intervention. This method is inductive and avoids using predetermined categories to allow for a better understanding of a phenomenon.

### Setting and Participants

The study took place at a university hospital that provides care and services to PLHIV. PLHIV were invited to participate if they were at least 18 years old and had been on ART for at least six months. Pregnant women, people with an uncontrolled psychiatric condition, and active intravenous drug users were excluded from the study. To be included in the qualitative section of the study, participants had to have completed all four VIH-TAVIE computer sessions and had to be able to share their experience in this regard with ease. Of the 99 PLHIV who received the virtual intervention, 73 completed all four sessions. For the purposes of this qualitative study, a convenience sample was used to explore experiences during the intervention. To achieve data saturation, 26 participants who completed all four sessions were recruited.

The study was approved by the Institutional Review Board of the Research Center of the Centre Hospitalier de l’Université de Montréal. Written consent was obtained from the participants after they were provided with a full verbal and written explanation of the study and advised that they could withdraw from the study at any time. Furthermore, the participants were assured that measures would be taken to ensure confidentiality throughout the research and protect their identity, including use of pseudonyms and modification of data collected during interviews (eg, workplace, physician’s name).

### Exposure to Intervention

VIH-TAVIE consists of four interactive computer sessions 20-30 minutes long. These are hosted by a virtual nurse who engages the user in a self-management skills-learning process. The sessions target different skills: self-assessment, motivational, problem-solving, emotion-regulation, and social. These enable PLHIV to integrate the therapeutic regimen in their daily routine, manage side effects, handle problem situations that might interfere with drug intake, interact with health professionals, and mobilize their social network. The sessions follow a predefined sequence in order to ensure a gradual transmission of skills [10].

**Figure 1:**
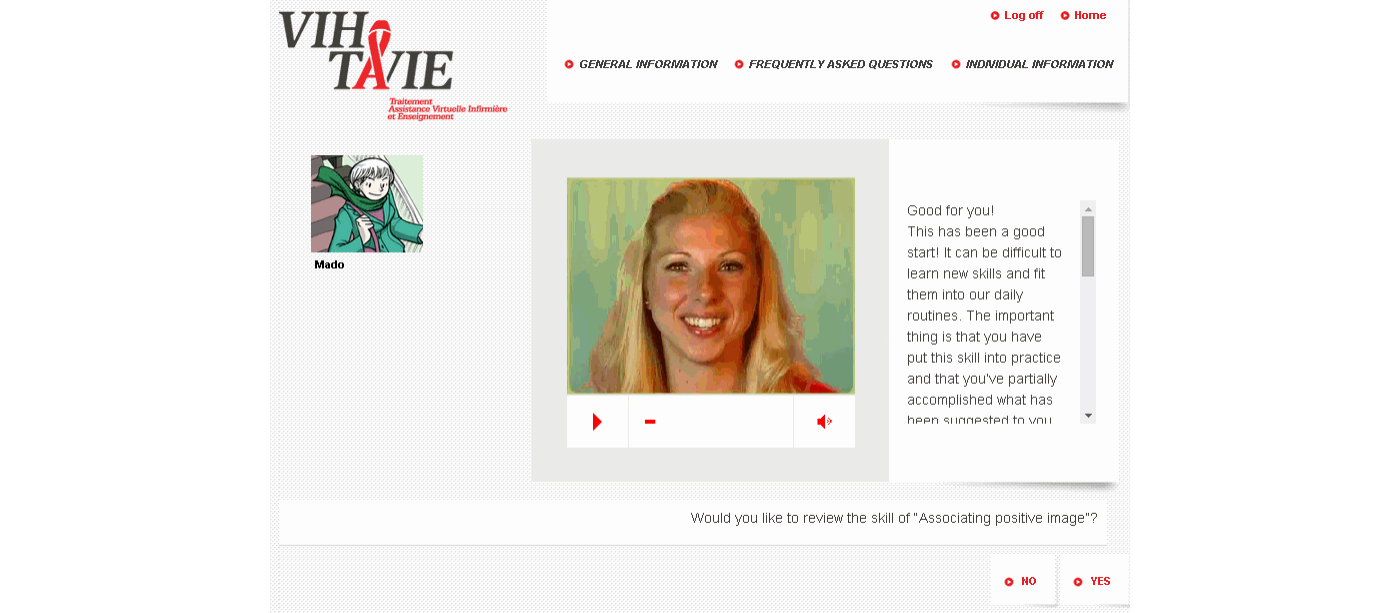
Screenshot of HIV Treatment, Virtual Nursing Assistance and Education (Virus de l’immunodéficience humaine - Traitement assistance virtuelle infirmière et enseignement) (VIH-TAVIE): interaction with the virtual nurse.

**Figure 2:**
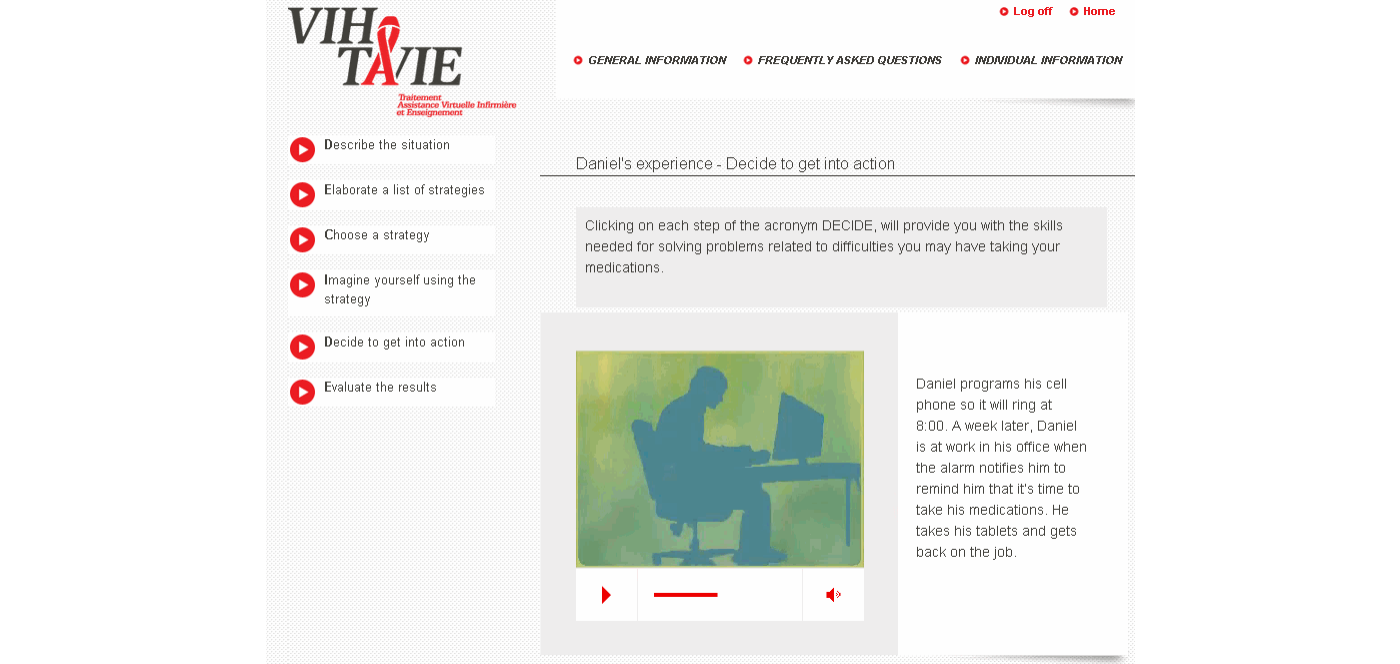
Experience of a person living with HIV (PLHIV) who has successfully overcome barriers. HIV Treatment, Virtual Nursing Assistance and Education (Virus de l’immunodéficience humaine - Traitement assistance virtuelle infirmière et enseignement) (VIH-TAVIE).

**Figure 3:**
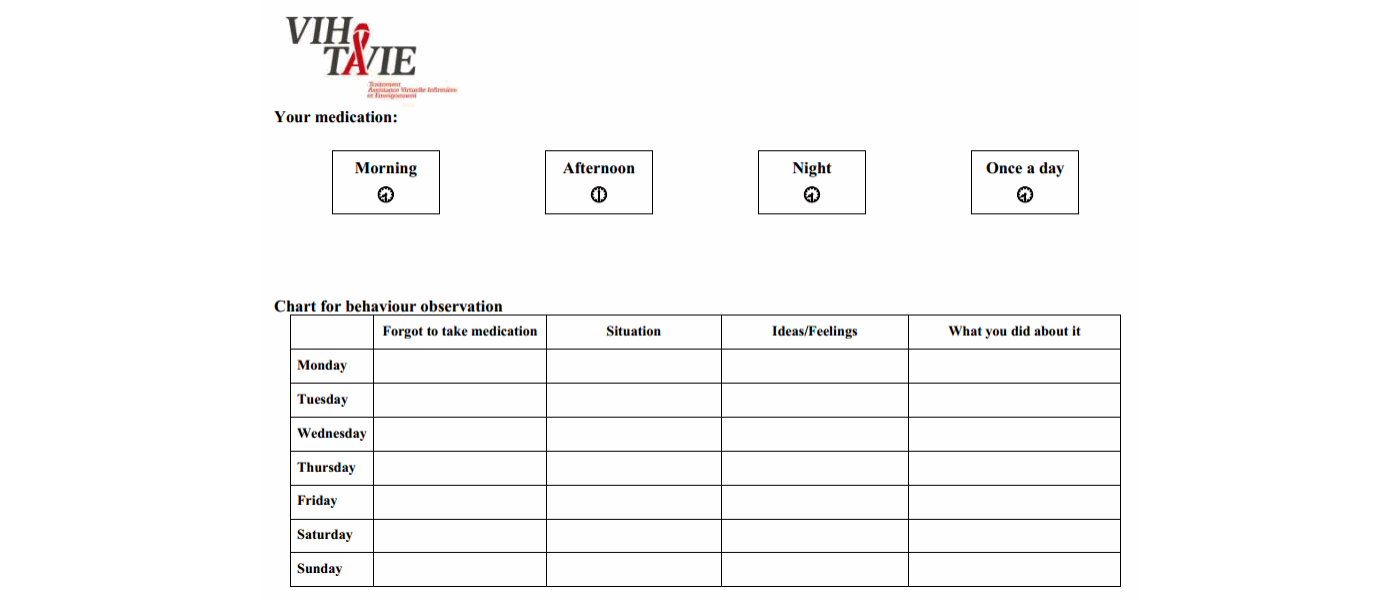
Chart for behavior observation. HIV Treatment, Virtual Nursing Assistance and Education (Virus de l’immunodéficience humaine - Traitement assistance virtuelle infirmière et enseignement) (VIH-TAVIE).

**Figure 4:**
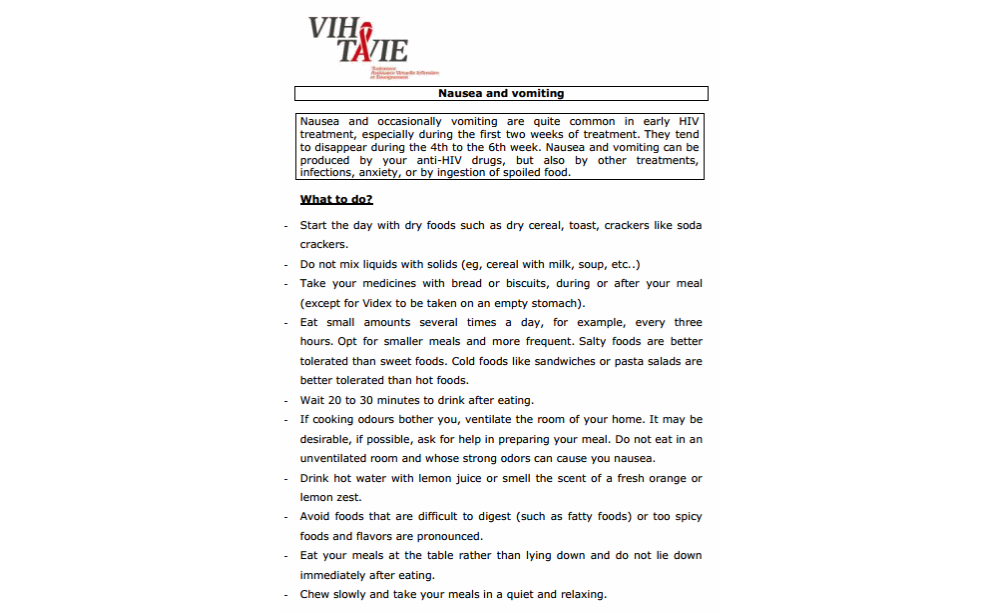
Advice on how to manage side effects. HIV Treatment, Virtual Nursing Assistance and Education (Virus de l’immunodéficience humaine - Traitement assistance virtuelle infirmière et enseignement) (VIH-TAVIE).

At the heart of the application is a virtual nurse coach who interacts with the user asynchronously (Figure 1 shows this). Over the course of the sessions, approximately 140 video clips of the virtual nurse are presented ranging in length from 10 to 90 seconds. She delivers tailored teaching following an algorithm based, for example, on the skills performed by the user or the user’s adherence level. Aside from the tailored teaching, the virtual nurse also refers to the experiences of other PLHIV who have coped successfully with situations similar to those of the user (Figure 2 shows this). During the sessions, the virtual nurse provides feedback and positive reinforcement on the user’s personal style and methods and on skills acquired. Each interactive session is distinct from the other in terms of message, strategies, skills, questions, and data entry. The intervention makes different tools available to help remember new information and skills and to have specifically tailored advice at hand. To this end, the tools in question can be printed out as PDF files. They are intended as support for using the newly acquired skills and, if desired, to keep track of progress. For example, a self-observation behavior chart (Figure 3 shows this) is available for users to identify and analyze the circumstances surrounding a lapse by answering questions such as: “What is the context in which the lapse occurred?”, “What was I doing?”, “Who was I with?”, “What was I thinking about?”, and “What can I do to prevent the situation from recurring?”. Some documents offer advice on how to manage side effects (Figure 4 shows this), while others are meant to facilitate drug intake. The latter tools recommend making use of positive image association and keeping a journal of side effects, and provide guidance regarding negative emotions, communication strategies, and social support. Finally, many other information documents can be accessed under the frequently asked questions (FAQ) section of the VIH-TAVIE Web interface [11].

This virtual nursing intervention is grounded in a disciplinary perspective, which is the McGill nursing model [12,13], and by extension, the strength-based approach [14]. With this approach, the person and his family are perceived as active participants in their health care and learn new ways to cope with difficult life events [12,13]. The virtual nurse supports the person in developing and reinforcing skills to manage difficulties by helping them to mobilize their strengths and their resources [15]. Bandura’s self-efficacy theory [16] was used, in particular to reinforce the individual’s confidence or capacity in managing their treatment/therapy, because self-efficacy was a main focus of the intervention. The virtual nurse aims to develop and reinforce self-management skills in individuals.

### Data Gathering

Individual semistructured interviews lasting on average from 30 to 40 minutes were conducted to allow participants to share their thoughts and feelings during the virtual nursing intervention. Few interviews lasted more than one hour. An interview guide was used to ensure all topics of interest were covered, including use/appropriation of technology, relevance of intervention, such as drug intake support, possible improvements to the intervention, and the interaction with the virtual nurse. The interview guide was developed by the research team that has an expertise on virtual intervention, with PLHIV, or qualitative research (JC, GR, PRG, AB). The questions reflected the objectives and the components of the virtual nursing intervention. The interview guide was refined during the data collection. The following are examples of questions asked: “Looking back on the VIH-TAVIE virtual intervention, what did you find least helpful?”, “What did you find most helpful?”, “What did you think of your contact and interactions with the virtual nurse?”, “Could you describe your experience during the computer sessions?”, and “Over the computer sessions, how did you feel?”. Allowing the participants to freely express their experiences in their own words contributed to obtaining a fuller description of the experiences. All the interviews were recorded and transcribed verbatim.

### Data Analysis

The data were analyzed using the Miles and Huberman’s method [17], whereby three activities were performed concurrently: (1) data reduction (data coding, summaries); (2) data display (in tables and text form); and (3) recontextualization of results. Each interview was coded by a principal coder. The codes were then validated by another person well acquainted with the project, and by means of consensus differences and discordances were resolved. The codes were examined for similarities and differences and grouped into categories. For instance, data associated with the virtual nurse constituted a group or category. Data capturing differences were classified under the same theme and an explanation for the differences was provided. In order to foster a more in-depth analysis, these similarities and differences were described in text form. The document was then sent to the rest of the research team for discussion. Various cycles of analysis and discussion took place, allowing for a refinement of the results and for extracting themes and subthemes relevant to the study’s purpose. These results were then recontextualized and held up against the existing literature. An iterative process was followed throughout the analysis, going back and forth between the transcripts and the interpretation of the results.

The research assistant and the research coordinator (GR) undertook the following measures to ensure the credibility and authenticity of the study, and to establish confidence in the study [18]: read the interviews multiple times; alternated between data collection and analysis; reached data saturation; and used peer review and held debriefing sessions with all the members of the research team (JC, AB, PRG). They took part in some discussions regarding data collected, analysis, and interpretation. Working documents allowed the research team to discuss the emerging themes, and to interpret the findings. This iterative process was helpful to draw a portrait of the experience of interest.

## Results

### Sample Characteristics

The sample consisted of 26 participants who completed all four sessions. Of these, 20 were men and 6 were women. Their mean age was 49 years (SD 8; range: 32-74) and 58% (15/26) had a high school education. There were 58% (15/26) that had annual income of less than CAD $15,000. They had been diagnosed with HIV 14 years earlier on average and had been on ART for a mean period of 10 years (see Table 1).

**Table 1:** Sociodemographic characteristics (N=26).

Characteristics		Participants’ data
Age (years), mean (SD), (min-max)		49, (8), (32-74)
**Gender, n (%)**		
	Male	20 (77)
	Female	6 (23)
**Ethnicity, n (%)**		
	Canadian	23 (88)
	Other^a^	3 (12)
**Marital status, n (%)**		
	Single	16 (62)
	Married or living as a couple	3 (12)
	Divorced/widowed	7 (27)
**Sexual orientation** ^b^ **, n (%)**		
	Heterosexual	10 (38)
	Homosexual	14 (54)
	Bisexual	1 (4)
**With children, n (%)**		
	No	18 (69)
	Yes	8 (31)
**Education level, n (%)**		
	Primary	1 (4)
	Secondary	15 (58)
	College	8 (31)
	University	2 (8)
**Annual income CAD, n (%)**		
	< $14,999	15 (58)
	$15,000-$24,999	7 (26)
	$25,000-$34,999	2 (8)
	> $35,000	2 (8)
**Employment status, n (%)**		
	Working/student	4 (15)
	Retired/unemployment insurance	2 (8)
	Welfare	13 (50)
	Other	7 (27)
**Living arrangements, n (%)**		
	Living alone	12 (46)
	With partner/family, friend	11 (42)
	Other (housing)	3 (12)
Years of HIV infection, mean (SD), (min-max)		14 (7.45), (0.50-26)
Years on ART, mean (SD), (min-max)		10 (5.67), (0.50-22)

^a^Other countries, South America 1, 4; others 2, 8

^b^Missing data, 1, 4

^c^Missing data, 1, 4

### Themes

The analyses yielded five major themes that describe the experience of having participated in a virtual nursing intervention in support of ART adherence. These themes and their corresponding subthemes are presented below.

### Exposure to the Virtual Nursing Intervention

Under this theme, the ergonomics of VIH-TAVIE and the presence of an actual nurse on site were explored as subthemes.

#### The Ergonomics of the Virtual Nursing Intervention

Though a small number of participants had technical difficulties associated with forgetting their log-in password, navigating the Web pages, or experienced stress when using a computer, the majority of the participants considered VIH-TAVIE to be user-friendly and enjoyable, and found it generally easy to navigate the interface,

I’m not familiar with computers, but it was great, it was explained very clearly[...]It went very well. It really wasn’t complicated.

Participant #5, 10 years of ART

The audiovisual content of the VIH-TAVIE intervention, such as the images, choice of avatar, video clips, and colors, was appreciated and made participation in the virtual intervention more captivating,

The colors are relaxing, they’re not aggressive. The colors, but also the choice of graphics was very nice[...]The window in which the virtual nurse is framed is not too big, it’s not full screen, which means you don’t feel invaded by her[...]Same with the graphics because they’re not overdone, they’re relatively simple.

Participant #23, 13 years of ART

#### Presence of an Actual Nurse on Site to Facilitate Transition to Virtual Mode

Several participants reported being reassured by the presence of an actual nurse on site before the start of the sessions, providing reassurance for the intervention with the virtual nurse. There was one person that underscored that having an actual nurse on site favored participant engagement in the intervention,

Had I been told simply to visit the site [VIH-TAVIE], I would have. But would I have stuck with the program to the end? I don’t know.

Participant #23, 13 years of ART

I just felt safe having the same nurse there all the time[...]Seeing how it was the same person welcoming me at the door and, then, there she was on the screen, well, it was reassuring.

Participant #26, 11 years of ART

Moreover, having a person nearby during the session was helpful for those who had little or no familiarity with computers, as they could refer to the on-site nurse when questions regarding the content emerged or they required help navigating,

That was very encouraging. Yeah. There’s always someone there with you if you have a problem or something, a button. She sorts it out.

Participant #9, 15 years of ART

### Virtual Nurse Humanizes Experience of the Computer-Delivered Intervention

Numerous persons had the feeling of being accompanied and of interacting synchronously with a nurse, as though they were using a Webcam. Yet, the interaction modality was asynchronous via video clips of the virtual nurse. The “human” and “warm” dimension of the intervention was emphasized by nearly all the participants*.* The virtual nurse was pleasant to listen to and watch. She provided clear explanations, tools, and tricks essential for drug intake,

It feels like you’re interacting. Uh, you don’t get the impression it’s a robot talking to you, you have the impression it’s someone explaining things clearly. I might add that the visuals of the virtual nurse give you the impression she’s real. She teaches you, she explains information, she gives, passes on information. 

Participant #15, 1 year of ART

I found the project...more personal with the virtual nurse than if it had just been a computer because it felt like I was with a person. I found that interesting. 

Participant #1, 7 years of ART

The whole thing of interacting with a virtual nurse, well, it makes me smile because...it’s less cold than simply reading information and interacting with a good computer program that responds to you[...]It’s less cold. So, finally, the reassuring side I was talking about, well, that’s it in a way. There’s someone there talking to you. It’s not a computer, it’s...it’s human. 

Participant #13, 13 years of ART

### Learner’s Experience of the Virtual Nursing Intervention

All of the participants appreciated the quality of the teaching delivered by the virtual nurse. In fact, the information presented in VIH-TAVIE was described as relevant and complete, as evidenced by the following transcript excerpt,

My experience with VIH-TAVIE was very worthwhile, very interesting, very rewarding[...]I must say in all honesty that it was very complete. Uh, it really covered all the bases. Uh[...]and, yeah, like it or not, in a way it forces you to question yourself all the time[...]

Participant #16, 16 years of ART

The majority of the PLHIV considered the questions easy to understand, but a minority found some questions lacked clarity or were repetitive. Some participants expressed feeling stressed while answering the questions and wanted to give their responses some thought to ensure their accuracy,

Trying to answer as best possible, as, uh, correctly as possible in terms of what corresponded to me[...]I felt that at times the answers to the questions didn’t come to me fast enough

Participant #12, 21 years of ART

This desire to provide the correct answer is a testament to the participants’ engagement in the virtual intervention.

Another positive point was the possibility of consulting the fact sheets (eg, skills summary, FAQ, advice regarding side effects) available on the VIH-TAVIE. These allowed the PLHIV to gain a better understanding of their side effects. These sheets were used as reference tools throughout the intervention and even after its completion. They allowed the PLHIV to consolidate, review, and sustain the learning achieved during the course of the sessions. The sheets were perceived as reassuring and helpful. This was the case even for participants who had been on ART for a long time. Since they were customized to their needs and accessible, the sheets empowered them to take charge of their condition.

I don’t know what I’d do without them [fact sheets]. I’d probably be over at my doctor’s all the time asking: “What do I do about feeling tired all the time? What do I do about my lack of appetite?” Instead, with all the fact sheets, I can get by on my own[...]I took all of the sheets so as to help myself. And, uh, these tools have been a great help. I mean, I was very happy to have them because, see, I’m still using them today.

Participant #12, 21 years of ART

Participants also appreciated the “novelty” of dealing with medication via a computer-delivered intervention. For some persons, learning to use a computer was seen as an accomplishment,

It’s a computer-based experience education-wise. Plus, you learn a bunch of stuff...working a computer, it’s important. You know, I didn’t know how to use a computer, but I learned

Participant #9, 15 years of ART

### Perceived Benefits Following Participation in the Virtual Nursing Intervention

#### Emotional Benefits

Completing the virtual nursing intervention allowed the participants to dampen their fears and their stress level regarding possible changes in medication. The intervention gave them confidence, reassured them, and encouraged them to continue with their ART. Acceptance of the disease and not feeling alone were also reported as other emotional benefits,

It cheered me up because I realized I wasn’t in the same, uh...I wasn’t the only one to have come down with this thing, it cheered me up. You know, I mean, listening to what she has to say about taking my medication[...]

Participant #12, 21 years of ART

It’s funny but knowing that I wasn’t the only one made me feel like, ok, I’m not here alone, I come here [VIH-TAVIE sessions] to improve my quality of life, like so many others who come here as well...So, how did I feel? I felt welcomed.

Participant #21

There was one participant that even referred to the intervention as “virtual therapy”. A sense of pride emanated from these experiences of having managed to regain control of their intake behavior. Here is an eloquent quote from a participant who reported being nonadherent prior to the intervention and for whom the benefits of having participated in the intervention were irrefutable, *Truth is, this experience saved my life.*

#### Using New Strategies/Skills

The participants reaped benefits from the information received and the tools and tricks proposed to foster optimal antiretroviral drug intake during the intervention. For most of them, the intervention was successful in answering their questions, particularly those regarding side effects, and directed them toward resources, such as community groups. The following example is illustrative of what various participants had to say in this regard,

It allows us, right, to develop strategies, ok, specific to each person, because each person has their own strengths and weaknesses[...]I found that the program offered me strategies, tools, gave me a lot of tools to make sure that I take my medication as prescribed[...]Plus, I have to say that the name of the program [the acronym VIH-TAVIE is the homophonic equivalent of “live your life” in French], uh...well, I find that it’s fitting in that the program allows us to live our life, uh, in a wholesome way and, yeah, fully and in good health. And to enjoy a good quality of life. 

Participant #15, 1 year of ART

Finally, the participants reported that, thanks to the intervention, they had gained a better understanding of the reasons for their lapses, acquired means to avoid them, and developed insight on how they took their medication. Many of them indicated that, prior to the intervention, they did not always grasp why they lapsed so often,

But [during the intervention] I thought about my medication intake, that’s for sure. About better ways of taking it. And it made me reflect also, uh, uh, on when I, of the times when I didn’t take it, why I didn’t take it. Because sometimes you don’t always give it much thought.

Participant #4, 11 years of ART

One of the benefits for PLHIV, therefore, was to have gained awareness of the importance of regular medication intake, which could translate into the application of strategies to optimize intake.

The PLHIV reported having used strategies proposed by the virtual nurse to manage side effects, to adopt a positive attitude toward medication, to try out visualization techniques, and to communicate with their health professionals (eg, physician). Many of the PLHIV reported having planned out a schedule for drug intake and having incorporated it in their routine,

Thanks probably, precisely to the information that I got at the computer, I was able right away to put it into practice, and then set and manage a suitable schedule to make it easier, precisely, to take my medication without it becoming a burden. 

Participant #16, 16 years of ART

### Relevance of the Virtual Nursing Intervention in Relation to the Medication Management Trajectory

#### Relevance Depends on Quality of Prior Antiretroviral Drug Intake

The participants described the relevance of VIH-TAVIE in relation to the quality of their drug intake behavior prior to the start of the computer sessions. In this regard, the higher the reported optimal drug intake, the lesser the effects of VIH-TAVIE appeared significant to participants. For example, for one participant who claimed to be adherent prior to the intervention, and, therefore, qualified his drug intake as regular, the intake behavior was maintained over time,

Before VIH-TAVIE? No different. I took my medication all the time, regular. I didn’t have any problems, none. 

Participant #5, 10 years of ART

For another participant who only rarely neglected to take his medication prior to VIH-TAVIE, there was some improvement,

I think that, before, I used to skip my medication a little more often. Now, I almost never do. It happened once this month, but it was only the first time in, in four months, I think. It’s not much. 

Participant #4, 11 years of ART

Moreover, for the three persons who previously reported being nonadherent, participation in the virtual nursing intervention enabled them to become aware of their situation. They realized that their behavior was not optimal owing to the numerous times they neglected to take their medication. Thanks to the intervention, they identified factors that interfered with their drug intake, such as precedence given to alcohol over medication and an unstable living environment (eg, poor housing). The intervention helped these persons overcome these difficulties and adopt optimal drug intake by reducing their lapses considerably,

Thanks to VIH-TAVIE, I take my medication now, it’s automatic[...]Look, I didn’t take my medication regularly before, I skipped doses all the time. Then I did four sessions at the computer, I saw the nurses[...]Well, bottom line is it works. That’s what matters.

Participant #8, 14 years of ART

Some participants considered that the virtual intervention helped them change other health-related behaviors,

Since doing those sessions...virtual, practical, written. Well, things have changed. Ok, I still like the occasional beer or a little toke. But what’s that? For me it’s nothing compared to before. I used to do coke every day. I’d shoot up, the whole shebang...Uh, everything available on the market I’d do. But now, those days are over. Done[...]Thanks to you, uh, to your programs and to...I read and reread, uh, my questions, the answers, everything in the kit that the nurse left me. 

Participant #10, 15 years of ART

#### Timing of HIV Treatment, Virtual Nursing Assistance and Education Relative to the Medication Management Trajectory

Most of the participants recognized the relevance and added value of the intervention as drug intake support. However, the large number of participants who had been on treatment a very long time felt that VIH-TAVIE would have more of an impact if offered to persons beginning therapy or having problems with drug intake,

I find the program effective for people who are starting medication rather than people who have been on it a long time[...]It’s effective for people who are either starting medication or have problems taking it[...]

Participant #4, 11 years of ART

For one participant who had been on ART for 8 years, VIH-TAVIE was timely, given that drug intake had its ups and downs,

Well, obviously [over the course of the VIH-TAVIE sessions I hoped] to improve my medication intake because I was lax in this regard[...]so it just so happened that the study come along at a time when, well, it’s not for nothing that I did this. Because, before, I was always quite regular and then I was like, there came a time where I was like, well, you become jaded.

Participant #17, 8 years of ART

## Discussion

### Main Results

Our study describes the experiences of PLHIV who took part in the VIH-TAVIE. There were five themes that emerged regarding their experience: (1) exposure to the virtual nursing intervention; (2) virtual nurse humanizes experience of the computer-delivered intervention; (3) learner’s experience of the virtual nursing intervention; (4) perceived benefits following participation in the virtual nursing intervention; and (5) relevance of the virtual nursing intervention in relation to the medication management trajectory.

Following coaching, the benefits perceived by the participants relate to skills and strategies that foster ART adherence. These include self-observation and lapse management skills, and other skills for integrating drug intake into the daily routine and managing side effects more effectively. Thus, the expected health behaviors and strategies/skills targeted by the intervention translated into gains/benefits for this service user group [11]. The coaching proposed by the virtual nurse was designed toward developing and consolidating skills requiring a sustained effort and greater engagement on the part of the user compared with interventions that primarily involve intake reminders. The development of skills during the intervention seems to contribute to improved self-efficacy as participants perceived greater self-confidence with antiretroviral drug intake going forward. As pointed out by Bandura [16], we believe that this sense of self-efficacy probably modified their cognitive processes and their emotional states. This phenomenon might explain why participants were less fearful of changes in medication, felt less isolation, and seemed to cope with the illness better.

In our study, it appears that the patients were able to engage and benefit from the intervention and perceived it as relevant. A theme that emerged was the intervention’s relevance or added value with respect to quality of drug intake and intake trajectory. According to the participants, the VIH-TAVIE intervention has a greater impact if offered to persons initiating therapy and to those with intake difficulties. This perception emphasizes the importance of meeting the “felt” needs of PLHIV and to ensure the timing of intervention. This information will be taken into account when the intervention is implemented, in order to target PLHIV more effectively.

While developing the intervention, it was important to simulate the interaction between the virtual nurse and the patient. To this end, a nurse played her professional role through an audiovisual modality, hence a “virtual nurse”. The idea was to develop an intervention that transcends the technology (machine) and allows for interacting with a virtual professional. The results suggest that the virtual nurse humanizes the experience of a computer-delivered intervention. In this regard, Barnard and Sandelowski [19] proposed to examine the nature of the “relationship” between two seemingly irreconcilable entities, such as technology (eg, reproductive techniques, imaging technologies) and humane nursing care. A parallel can be drawn between ICT-assisted interventions such as VIH-TAVIE and the humanization of care. According to these authors, the determinant of whether technology dehumanizes and depersonalizes is not the technology per se, but the manner in which it is employed and operationalized in specific contexts, the meaning attributed to it, and how individuals or cultural groups define what is “humane”. Sandelowski [20] discussed the notions of presence (being there physically with patients) and place (space), which must be redefined for nurses in the era of technology (cyberspace). Nurses and media designers wish to create a comfortable environment to interact that is intimate and “real” [21]. It is in the nurses' interest to create a climate of shared space where the sense of being with patients is delivered through the technology.

The intervention was developed as an adjunct to clinical care. The study participants made it clear that it was important for the virtual intervention to fall within a care trajectory. With respect to the first theme, “exposure to the virtual intervention”, they expressed that the on-site nurse facilitated the transition to the virtual nurse and this face-to-face contact made them feel at ease with the virtual nurse. There was one participant that found that being accompanied by an actual nurse on site fostered engagement in the intervention. In a systematic review by McDermott and While [22], the utilization of computer technology to promote self-management of chronic illness in health care settings was found to hold great promise, with the potential to change both health behaviors and clinical outcomes among chronically ill patients.

The VIH-TAVIE virtual intervention is a customized health program that provides tailored content, but also virtual coaching and educational tools to develop the skills required to enact health behaviors. The third theme, “learner’s experience”, brought out the PLHIV’s appreciation for the quality of the content and the relevance of the information received, as in managing side effects. In a review by Linn et al [23] of the effectiveness of ICT-assisted interventions in enhancing patient adherence to prescribed long-term medication (n=13), most of the interventions reviewed were moderately or highly sophisticated tailored interventions.

### Implications for Research and Practice

We propose that further research be done using a mixed-method design to capture the overall value of this type of virtual nursing intervention in different contexts. Based on the participants feedback, it would be relevant to evaluate the efficacy of VIH-TAVIE among PLHIV who have difficulties with their antiretroviral intake (ie, those who are referred by their health care professionals; and those who have a detectable viral load and high level of CD4 count) as well as those who begin the antiretroviral medication.

Since we plan to implement VIH-TAVIE for all PLHIV and to offer them an additional and complementary service as support in their treatment taking behavior, our qualitative results will be taken into account in implementing strategies. In doing so, VIH-TAVIE must be embedded into clinical practice, as part of the routine of health care professionals.

### Limitations

Despite the benefits that users experience from receiving the VIH-TAVIE virtual nursing intervention gleaned from this study (ie, receiving customized accompaniment via a virtual nurse asynchronously), its single focus on participants who had completed all four computer sessions is a limiting factor.

The use of a maximum variation sample to explore various experiences during the intervention, namely, to collect data among participants who completed one, two, or three computer sessions would have greatly enhanced the study. The experience of a participant who completed only one session is likely different from one who finished the entire virtual nursing intervention. In addition, some participants completed the interview immediately after the fourth computer session of VIH-TAVIE, while other PLHIV did the interview after full participation in the study (three months after completion of the fourth computer session). It appeared easier for the participants to report on their experiences in a virtual nursing intervention immediately following the final computer session. For the purposes of this qualitative study, we were not guided by the principle of data saturation. We chose participants according to a convenience sample, which might be a limitation of our study. In return, we are confident that data saturation has been reached because no new information was obtained [24]. The motivation for using this methodology was to gain a better understanding of the experience of the persons who engaged in the intervention. In addition, this user group made up the majority of the sample (74%, 73 of 99 participants).

### Conclusions

In summary, by analyzing the participants’ experience, we reveal that they found the intervention’s content and format appropriate. In their eyes, the virtual nurse humanized the experience and helped them acquire new skills for achieving optimal antiretroviral drug intake. Moreover, this experience seemed to modify the participants’ cognitive and emotional processes and to boost their self-confidence with respect to drug intake. Finally, the results seem to underscore the importance of offering the intervention to persons who have more problems with drug intake or who are just beginning ART. In closing, advances in ICT present an opportunity to offer adjunctive services that fit within the continuum of care. ICT constitute an indispensable means of meeting the current challenges of care accessibility and continuity.

## Abbreviations

ART: antiretroviral therapy
FAQ: frequently asked questions
HIV: human immunodeficiency virus
ICTs: information and communication technologies
PLHIV: persons living with HIV
SOC: standard of care
VIH-TAVIE: HIV Treatment, Virtual Nursing Assistance and Education (Virus de l’immunodéficience humaine - Traitement assistance virtuelle infirmière et enseignement)
